# Comparative and functional genomics of the ABC transporter superfamily across arthropods

**DOI:** 10.1186/s12864-021-07861-2

**Published:** 2021-07-19

**Authors:** Shane Denecke, Ivan Rankić, Olympia Driva, Megha Kalsi, Ngoc Bao Hang Luong, Benjamin Buer, Ralf Nauen, Sven Geibel, John Vontas

**Affiliations:** 1 Institute of Molecular Biology & Biotechnology, Foundation for Research & Technology Hellas, 100 N. Plastira Street, 700 13, Heraklion Crete, Greece; 2 Department of Chemistry and Biochemistry, Mendel University in Brno, Zemedelska 1, 613 00 Brno, Czechia; 3 CropScience Division, Bayer AG, R&D Pest Control, D-40789 Monheim, Germany; 4 Laboratory of Pesticide Science, Department of Crop Science, Agricultural University of Athens, Athens, Greece

**Keywords:** ABC transporters, Comparative genomics, Gene family evolution, RNAi, Arthropod

## Abstract

**Background:**

The ATP-binding cassette (ABC) transporter superfamily is comprised predominantly of proteins which directly utilize energy from ATP to move molecules across the plasma membrane. Although they have been the subject of frequent investigation across many taxa, arthropod ABCs have been less well studied. While the manual annotation of ABC transporters has been performed in many arthropods, there has so far been no systematic comparison of the superfamily within this order using the increasing number of sequenced genomes. Furthermore, functional work on these genes is limited.

**Results:**

Here, we developed a standardized pipeline to annotate ABCs from predicted proteomes and used it to perform comparative genomics on ABC families across arthropod lineages. Using Kruskal-Wallis tests and the Computational Analysis of gene Family Evolution (CAFE), we were able to observe significant expansions of the ABC-B full transporters (P-glycoproteins) in Lepidoptera and the ABC-H transporters in Hemiptera. RNA-sequencing of epithelia tissues in the Lepidoptera *Helicoverpa armigera* showed that the 7 P-glycoprotein paralogues differ substantially in their tissue distribution, suggesting a spatial division of labor. It also seems that functional redundancy is a feature of these transporters as RNAi knockdown showed that most transporters are dispensable with the exception of the highly conserved gene *Snu*, which is probably due to its role in cuticular formation.

**Conclusions:**

We have performed an annotation of the ABC superfamily across > 150 arthropod species for which good quality protein annotations exist. Our findings highlight specific expansions of ABC transporter families which suggest evolutionary adaptation. Future work will be able to use this analysis as a resource to provide a better understanding of the ABC superfamily in arthropods.

**Supplementary Information:**

The online version contains supplementary material available at 10.1186/s12864-021-07861-2.

## Introduction

The ATP-binding cassette (ABC) superfamily is one of the best studied gene groups in biology [1]. The majority of proteins within the superfamily act as transporters, shuttling a wide variety of endogenous compounds and xenobiotics across lipid membranes. Others, despite their nomenclature, lack transmembrane domains and play other essential cellular functions [2]. Each functioning transporter is made up of two highly conserved nucleotide binding domains (NBDs) and two less conserved transmembrane domains (TMs), which may be found in a single polypeptide (full transporter) or split into multiple subunits that must unite (half transporter) to form functional proteins. They are present in a diverse set of taxonomic lineages from “microorganisms to man” [3]⁠ and play a variety of physiological functions. Individual ABCs can be grouped into nine families (named ABC-A through ABC-I) based on the sequence homology in their conserved NBD. Although there exists substantial variation within these subgroups, family members share some common allocrites and functions across a range of organisms [4]. Classification of genes into a particular ABC family can therefore be used as a starting point to investigate potentially interesting genes in non-model organisms.

Less is known about the ABC superfamily in arthropods compared to other taxonomic groups such as mammals or bacteria [4]. However, there has been an increasing effort to understand the molecular biology of arthropods for several reasons. The arthropod phylum is the most diverse of the multicellular eukaryotes and is frequently studied in order to gain fundamental evolutionary insights [5]⁠. Additionally, many arthropod species are pests, having substantial impact in both agriculture and public health. Pesticides including small molecules and protein based biopesticides are the most common way to control these pests, and ABC transporters are thought to play a key role in pesticide biology [6, 7]. Xenobiotic transporting roles have been suggested for the ABC-A, ABC-B, ABC-C, and ABC-G families families [6, 8]⁠, while members of the ABC-C and ABC-A family often serve as targets for crystal toxins derived from *Bacillus thuringiensis* [9].

One of the best studied ABC families is the ABC-B clade which consists of both full and half transporters that act on an array of substrates including neutral and cationic amphiphilic compounds [10]⁠. ABC-B full transporters are more commonly known as multidrug resistance proteins (MDRs) or permeability glycoproteins (P-glycoproteins; P-gps) because of their ability to excrete xenobiotic compounds. Human P-gp activity was inhibited by pesticides such as (cypermehtrin, fenvalerate, endosulfan and methyl parathion), suggesting that these compounds may be substrates for this protein [11]⁠. P-gp orthologues in insects have been shown to underpin pesticide resistance in cases such as *Heliothis virescens* [12]⁠ and *Drosophila melanogaster* [13]⁠. In other cases, genetic manipulation of P-glycoproteins increased or decreased the toxicity of a pesticide in susceptible backgrounds [14, 15]⁠. However, the genomic trends of P-gp have not been thoroughly investigated.

The ABC-H transporter family is also particularly interesting due to its distribution across species. This family is present in arthropods and a limited number of other species scattered across the tree of life [6]⁠. The majority of arthropods contain fewer than 6 ABC-H members, but larger numbers were found in two non-insect arthropods *Tetranychus urticae* and *Daphnia pulex* along with two hemipteran species (*Bemisia tabaci* and *Diaphorina citri*) [16–19]⁠. Structurally, ABC-H proteins are half-transporters and show the same inverted domain architecture as the ABC-G family. The functional characterization of these proteins has been pioneered in *Drosophila* where the ABC-H transporter *Snustorr* (*Snu*) was localized to the integument and shown to transport cuticular hydrocarbons [20]. RNAi knockdown of *Snu* orthologues in other arthropods has led to lethality due to desiccation, suggesting a similar function [21, 22]⁠, but little information exists on other members of the ABC-H family.

Insights into the evolution of a gene family can highlight expansions or contractions which may suggest functional adaptation. Previous studies have taken such an approach by comparing ABC families across arthropod species, albeit on a limited number (7) of species [6]⁠. In the intervening years the number of sequenced insect genomes has greatly increased thanks in large part to the i5k project and a variety of independent labs sequencing various arthropods from many taxonomic groups [23]. This has led to an avalanche of publications which manually annotate the ABC family of a species using a variety of different methodologies [17, 24, 25]⁠. So far, there has been little effort to systematically compare the ABC transporter superfamily in arthropods. Such a comparative genomic analysis of the ABC superfamily was previously accomplished in plants, [26]⁠ and a more recent study has considered the evolution of the Solute carrier (SLC) transporter superfamily in arthropods [27]. To the authors knowledge, only one study has taken this approach in arthropod ABC transporters, but this work did not distinguish between ABC families and rather considered only the total number of ABCs in a species [28]⁠.

Here, we extend the knowledge of the ABC transporter superfamily in arthropods through comparative and functional genomics. First, we designed and implemented the *ABC_scan* algorithm to identify and classify ABC transporters from the predicted proteome of a species. Comparing the family sizes of different ABC transporter families showed substantial expansions in the lepidopteran ABC-B full transporters (ABC-BF; P-glycoproteins) and the hemipteran ABC-H transporters. Transcriptomics of relevant epithelial tissues from a model Lepidoptera *Helicoverpa armigera* and an RNAi screen in a model Hemiptera *Nezara viridula* were then used to further probe the properties and functions of these expansions.

## Materials and methods

### In silico identification of transporters

**Fig. 1:**
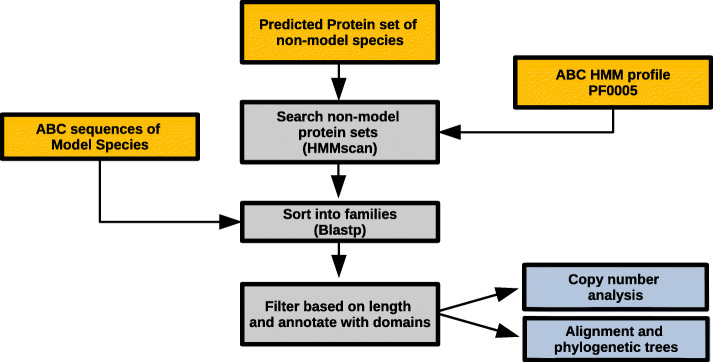
*ABC_scan* pipeline schematic. The *ABC_scan* pipeline can be separated into inputs (orange), actions (grey), and outputs (blue). Predicted protein sets for a given species are fed into the pipeline. In the first step the protein sets are searched with the ABC transporter (PF00005) HMM profile. Next, candidate transporters are sorted into families by blasting against a database of known ABC transporter proteins from *H. sapiens, T. castaneum, D. melanogaster*, and *T. urticae*. Lastly, results are filtered based on their amino acid length and annotated with corresponding metadata before outputting the predicted ABC set in tabular and fasta format

Putative ABC transporters were identified in non-model arthropod species using an *in silico* pipeline dubbed *ABC_scan* that made use of previously annotated ABC transporters to search the predicted protein sets of target species (Fig. 1; https://github.com/shanedenecke/ABC_scan.git). Unigene protein sets containing a single amino acid sequence per gene were obtained primarily from OrthoDB supplemented with other sources (Table S1). As overestimates or underestimates of the total number of proteins in a species would bias our overall results, we used the Benchmarking Universal Single-Copy Orthologs (BUSCO) method to remove lower quality protein sets [29]⁠. Unigene protein sets for each species were analyzed with BUSCO using the arthropod odb10 lineage (-l arthropod_odb10) under the proteins setting, and species with a single copy completeness score of below 80 % were excluded from the analysis.

The Hidden Markov Model (HMM) profile corresponding to the ABC nucleotide binding domain (PF00005) was retrieved from PFAM (http://pfam.xfam.org/fa3zmily/PF00005.23) and used to search predicted protein sets of different arthropod species with the HMMER package v3.2.1 [30]⁠. Candidate transporters with an e-value of less than 10 were kept as potential candidates. These sequences were then used as BLASTP [31] queries (e-value cutoff of 1e-5) against a database comprising proteomes of 4 species (*Homo sapiens, Tetranychus urticae, Drosophila melanogaster* and *Tribolium castaneum*) with well annotated ABC sets including family (A-H) annotation [19, 32, 33]⁠.

Classification of each candidate sequence into an ABC family was performed based on their most significant BLAST hits. If 4 out of the top 5 hits were all the same family or the top 3 blast hits were all the same family the candidate was considered part of that family. Alternatively, a candidate was sorted into the family of its top hit if this protein was an ABC transporter and possessed an e-value greater than 5 orders of magnitude lower than the next best hit. Candidates not meeting these criteria were not considered ABCs and excluded from this study. Proteins showing ambiguity between the ABC-B full and half transporter subfamilies were categorized based on their number of nucleotide binding domains; ABC-BH proteins had one NBD whereas ABC-BF transporters had two or more. The ABC-I family was recently proposed in insects [25]⁠, but it was excluded in this study because it was not annotated in any of the model insect proteomes on which the analysis depends.

### Analysis of family size over evolutionary time

The size of each ABC family (A-H) in each species included in the final analysis were compared in order to identify expansions and contractions over evolutionary time. This was accomplished by first grouping species by their taxonomic group and using a Kruskal-Wallis statistical test with a Bonferroni correction to identify groups with significantly larger or smaller ABC families. This strategy was implemented using the “kruskal” function from the “agricolae” package in R, which calculated p-values reflecting significant differences among taxa and categorized taxa into statistical groups based on their average family sizes.

Family sizes within taxonomic groups were further explored with the Computational Analysis of gene Family Evolution v5 (CAFE; [34]; https://github.com/hahnlab/CAFE5), which uses phylogenomics and family sizes to estimate the timing of gene family evolution. Family sizes for each ABC family in each species were estimated using the ABC_scan pipeline described above. Ultrametric phylogenies were generated for selected subclades of arthropods (Table S2) by first gathering 1:1 orthologues using Orthofinder v2.3.11 [35]⁠⁠. Amino acid sequences from each ortholog group were then aligned with MAFFT [36]⁠ and trimmed with Trimal v1.4 [37]⁠ using default parameters for the former and the “automated1” algorithm for the latter. These trimmed alignments were then concatenated to form a single sequence for each species and used as an input to RAxML-NG v0.9.0 [38]⁠ using the “LG + G8 + F” model for a maximum of 200 bootstraps. Trees were then calibrated using the “chronos’’ function of the “ape” R package v5.4-1 using a discrete model and known evolutionary divergences from a previously published study [39]⁠. These trees were then rooted using the “root” function from the same package with designated outgroups (Table S2). All trees were visualized in R with the ggtree package v2.2.4 [40]⁠.

The same procedure (MAFFT, Trimal, RAxML-NG) was used to make protein level phylogenetic trees of selected transporters. For the ABC-B full transporters, sequences from *Bombyx mori, Spodoptera frugiperda, Nezara viridula, Danaus plexipus, and Papilio polytes* were used. In the ABC-H family *N. viridula, Bemisia tabaci, Daphnia pulex, T. urticae, Nilaparvata lugens, Myzus persicae, Rhodnius prolix*, and *Daktulosphaira vitifoliae* were used. Branches with below 50 % bootstrap values were collapsed as polytomic nodes.

### *Helicoverpa* dissections and RNA-seq

A population of *H. armigera* was obtained from Bayer Crop Sciences and reared in the laboratory for several generations at the Institute of Molecular Biology and Biotechnology (Greece). Larvae were maintained on artificial diet, and adults were fed 10 % sugar solution. All individuals were kept at 16:8 light:dark at 26 °C. Dissections of midgut, Malpighian tubules, and central nervous system tissues were performed on L5 larvae using forceps under RNAse free phosphate-buffered saline (PBS) on ice, and RNA was extracted with the GeneJet RNA purification kit (Thermo Scientific). RNA samples were shipped on dry ice to the Macrogen Sequencing facility (Seoul, Korea) and libraries were prepared with the Illumina TruSeq stranded mRNA kit. Libraries were sequenced with the Illumina HiSeq 2500 platform with 100 bp paired end reads.

Raw FastQ reads were first analyzed using FastQC [41]⁠, and the FastP program was used for adapter removal and trimming of the sequences using the “-g” flag to remove poly G sequences and the “–detect_adapter_for_pe” flag to remove paired end adapters [42]⁠. Pair end reads were mapped to the *Helicoverpa armigera* genome (GCF_002156985.1) using HISAT2 v2.1.0 [43] with default parameters. The mapped reads were further sorted using Samtools v1.10-38 [44] and FeatureCounts v2.0.0 was used to quantify gene expression using the official gene set GFF file (https://data.csiro.au/dap/landingpage?pid=csiro%3A23812). All raw counts were normalized to transcripts per million (TPM) for cross sample comparisons.

### *Nezara viridula* maintenance

The eggs of *N. viridula* were obtained from Bayer AG and reared in large mesh cages. Eggs, nymphs, and adults were kept under controlled conditions at 23 ± 1 °C with a 12:12 light:dark photoperiod. The diet of nymphs and adult insects was the same: organic beans, carrots, as well as sunflower seeds.

### dsRNA synthesis

cDNA from nymphs was used as a template for dsRNA synthesis. Amplicons of a 300-400 bp region of selected genes was amplified by PCR with Phusion® DNA Polymerase Kit (New England BioLabs). The forward and the reverse primers had a T7 promoter sequence at their 5’ end (Table S3). PCR conditions were 98 °C for 30 s for an initial denaturation, followed by 35 cycles of denaturation at 98 °C for 10 s, annealing at 64 °C for 20 s, extension at 72 °C for 20 s, and a final step of extension at 72 °C for 5 min. After PCR, the amplicons were purified using NucleoSpin® Gel and PCR Clean-up kit (Macherey-Nagel). Purity was checked on 1.5 % agarose gels. Purified DNA was then used as a template for in vitro dsRNA synthesis using the HiScribe™ T7 High Yield RNA Synthesis Kit (New England BioLabs). Extraction (Phenol:Chloroform) and ethanol precipitation was performed according to the manufacturers protocol (New England BioLabs). The resulting dsRNA was diluted in injection buffer at a final concentration of 2 µg / µL.

### *Nezara* nymph injection

Nymph injections were performed using a protocol established in a previous publication [45]⁠. Three days after hatching, nymphs were transferred to agar step block that had been incubated on ice for 10 min. Nymphs were lined up with their dorsal side facing the agar and, were incubated ± 5 min on ice. Approximately, 20 nL of 2 µg/µL dsRNA was injected to each nymph using an IM 300 Microinjector (Narishige, Japan). In total, eight different genes were tested, and ds-LacZ was used as a negative control. The injected nymphs were transferred to separate boxes under laboratory conditions and mortality was observed by the naked eye between 3 and 10 days after injection. Mortality was measured by Schneider-Orelli’s formula. The experiment was performed with 75 nymphs in each repetition. Of these 25 nymphs (5 replicates of 5 nymphs) were collected for RNA extraction 5 days after injection.

For qPCR, TriZol was used to extract RNA of 5 nymphs, of which 1 µg was used for cDNA synthesis using the oligo-dT primer and the reverse transcriptase kit from EnzyQuest (Heraklion, Greece). SYBR-Green master mix (Invitrogen) was then used for quantification with primers specific to each target gene or the RP60 and 18 s housekeeping genes (Table S3) by employing the ΔΔCt method. All reactions were set up with .5µM of each primer and run on a CFX Connect (BioRad) machine. Efficiency estimates for all primer pairs were obtained with a 5-fold dilution series, and at least 3 biological replicates were performed for each gene.

### Data availability

The full source code for the ABC_scan pipeline is available on GitHub (https://github.com/shanedenecke/ABC_scan.git), and a web application for users to identify ABC transporters from fasta protein files is available at (http://chrysalida.imbb.forth.gr:3838/ABC_scan/). The full analysis for this study (including BUSCO, CAFE etc.) is also available on GitHub (https://github.com/shanedenecke/ABC_ID_SCRIPTS.git). Raw RNA-seq data from H. armigera were submitted to the sequence read archive (SRA) under the bioproject accession (PRJNA719695).

## Results

### Annotation of ABC transporters across arthropod species

In order to understand and study the evolution of the ABC transporter superfamily across arthropods, we designed the *ABC_scan* pipeline to identify and classify ABCs in 193 non-model species (excluding the 4 model species used in the search algorithm) with sequenced genomes derived from sources such as OrthoDB, NCBI, and others. Of these, 158 had BUSCO single copy completeness scores of > 80 % and were analyzed with the *ABC_scan* pipeline (Table S1). A total of 8,803 predicted total transporter sequences were identified averaging ~ 55/species, ranging from a low of 34 in the green orchard bee *Euglossa dilemma* to a high of 132 in the springtail *Folsomia candida* (Figure S1, Table S1). The general quality of the *ABC_scan* identification algorithm was assessed by comparing the numbers of predicted transporters against numbers previously reported in the literature. Discrepancies between the *ABC_scan* pipeline implemented in this study and literature derived predicted transporters ranged from a 7 % overestimation to an 8 % underestimation with a mean deviation of 0.3 % (Figure S2; Table S4), suggesting that the *ABC_scan* pipeline was generally suitable for predicting ABC gene sets across arthropods. A publicly facing web application was also developed using R-shiny where users can upload a predicted protein set for a species (fasta format), which can then be scanned for ABC transporters (http://chrysalida.imbb.forth.gr:3838/ABC_scan/).

**Fig. 2:**
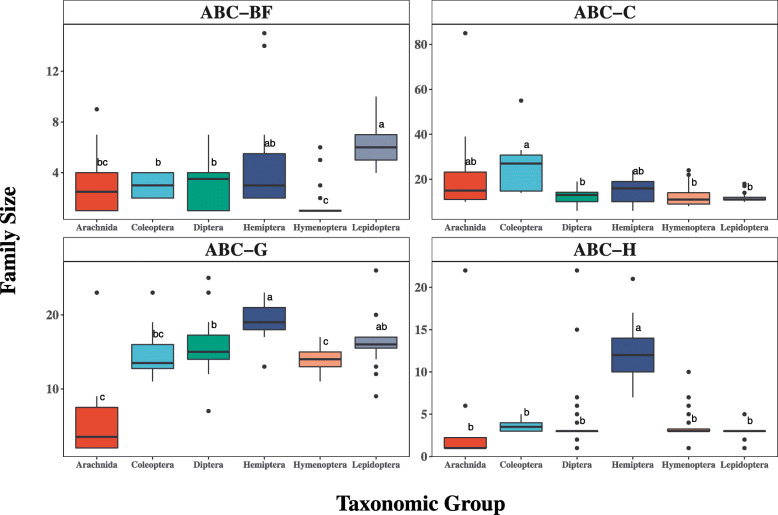
ABC family size variation across taxa. A comparison was made of all ABC family sizes (y-axis) broken down by both taxonomic order (x-axis) and family (panels). Orders are color coded, and boxplots include a horizontal black bar for median, boxes for upper quartiles, dots for outliers. Lower case letters above the boxes signify statistical groups generated by the Kruskal-Wallis test. An interactive version of the figure can be found on the R-Shiny Webb application (http://chrysalida.imbb.forth.gr:3838/ABC_scan/)

A preliminary understanding of how ABC families differed among arthropod lineages was gained by dividing species into taxonomic groups and comparing their family sizes. Small amounts of variation were observed among the ABC-BH, ABC-D, ABC-E, and ABC-F families (Figure S3; Table S5). While there were statistical differences among the ABC-D family, the small magnitude of the change made it difficult to explore in detail. The largest differences from the Kruskal-Wallis test comparisons were concentrated in the ABC-A, ABC-BF, ABC-C, ABC-G, and ABC-H families (Fig. 2; Table S5). The ABC-G family was notable in that it appeared much smaller in all Arachnids (median of 3.5 genes) versus a median of 15 genes for all species combined. The arachnid *T. urticae* is an extreme outlier with 23 ABCG genes. The ABC-C family was significantly higher in Coleoptera with a median of 27 genes compared to 13 found among all arthropods. This expansion appeared to be specific to the Cucujiformia species such as *T. castaneum* and *Leptinotarsa decemlineata* (Figure S4). However, the most striking expansions supported by the greatest number of species were among lepidopteran ABC-B full transporters and hemipteran ABC-H transporters (Fig. 2). Therefore, these two cases were explored in further detail.

### ABC-B Full Transporters in Lepidoptera

**Fig. 3:**
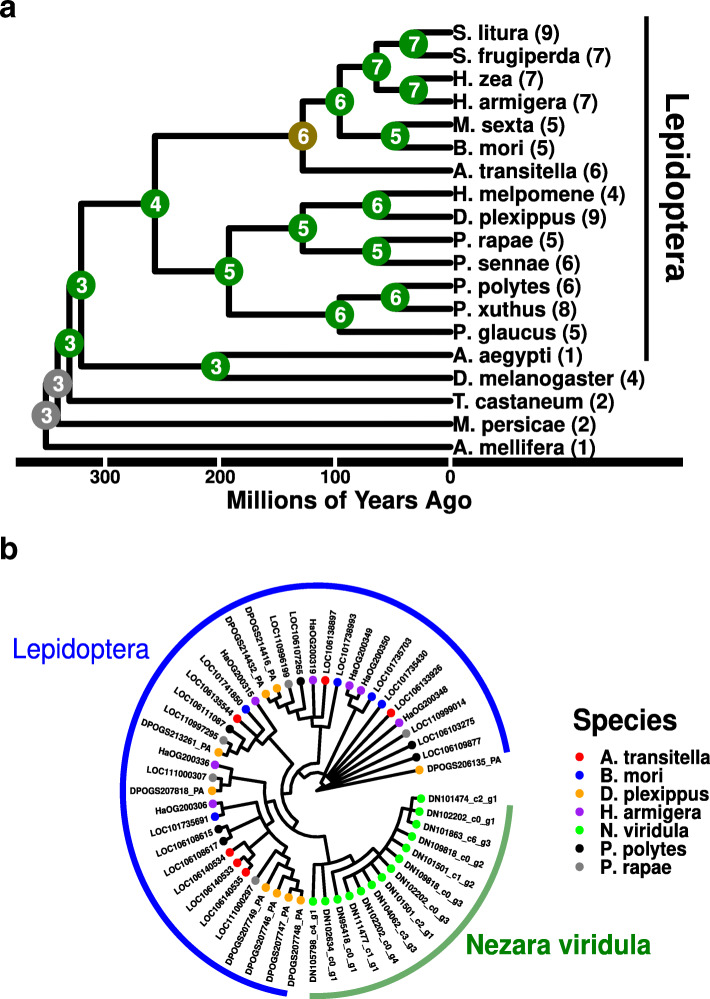
The ABC- B full transporters in Lepidoptera. **a** The evolution within the ABC-B full transporter family was analyzed with CAFE. Each tip of the tree represents a species mostly of Lepidoptera. The numbers present next to each tip correspond to the number of predicted ABC- B full transporters, while the node numbers correspond to CAFE predictions for ABC-B full transporter family sizes. Color coding of the nodes signifies bootstrap support with > 90 % = Green; 70–90 % =Yellow; <70 % = Red; NA = Gray. **b** A phylogeny of a subset of ABC- B full transporters was made in order to understand the relationship of these transporters among Lepidoptera and other groups. The majority of P-glycoproteins from Lepidoptera do not show species specific expansions suggesting that an expansion occurred common to all Lepidoptera. In contrast, the clustering of the *N. viridula* P-glycoproteins suggest a distinct origin for this expansion

The ABC-B family is known to play roles in physiological homeostasis and drug transport. To study the evolutionary history of the Lepidoptera expansions, we built phylogenetic trees at the species and gene level. The Lepidoptera expansion of ABC-BF transporters appeared to take place in all members of this clade, and CAFE analysis did not identify any significant expansions or contractions within the Lepidoptera clade (Fig. 3A). Further characterization of the ABC-BF expansion was performed by creating a phylogeny of all ABC-BF transporters from 6 Lepidoptera species and *N. viridula*, which is not a Lepidoptera but has a large number of ABC-BF transporters. Among the Lepidoptera there was no observed species specific clustering of transporters while all *N. viridula* ABC-BF transporters grouped together (Fig. 3B). This suggests that the ABC-BF expansion in Lepidoptera likely occurred at the beginning of the Lepidoptera lineage.

**Fig. 4:**
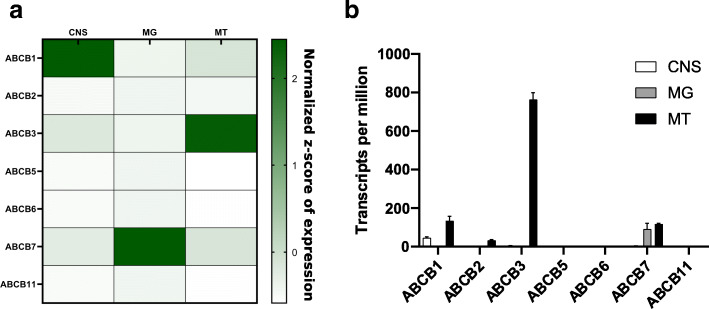
Expression of the ABC-B full transporters in. *H. armigera*. **a** Heatmap showing differential expression of the ABC-B paralogues in different tissues of fifth larval stage H. armigera based on transcript per million values, TPM (Z-transformed) values obtained using RNA-seq. Where CNS = central nervous system, MG = midgut and MT = Malpighian tubules. Each box represents the mean value of four biological replicates (*n* = 4). **b** Bar graph showing the total expression of each ABC-BF gene in each tissue (CNS = white, MG = grey, MT = black)

Transcriptomic analysis on the midgut, Malpighian tubules, and central nervous system of the model Lepidoptera *H. armigera* was employed to further characterize the ABCB full transporters. 7 full transporters were identified in our pipeline which were in agreement with a previous study and were named in accordance with the official gene set nomenclature (Fig. 4; Table S6) [46]⁠. In the Malpighian tubules several paralogues showed noteworthy expression measured in transcripts per million (TPM) including *HaABC-B3* (761.85 TPM), *HaABC-B1* (131.68 TPM), *HaABC-B7* (116.46 TPM), *HarmABC-B2* (31.24 TPM). Interestingly, in the midgut tissue only one gene, *HaABC-B7* (89.34 TPM) was highly expressed. Similarly, the central nervous system expressed only *HaABC-B1* (44.62 TPM) at high levels. The paralogues *HaABC-B5*, *HaABC-B6*, *HaABC-B11* all showed minimal expression in all the tissues sampled. In total, the variable expression indicates that different P-glycoprotein paralogues show highly divergent expression patterns and that each epithelial tissue sampled has a different paralogue that predominates.

### The ABC-H Family in Hemiptera

**Fig. 5:**
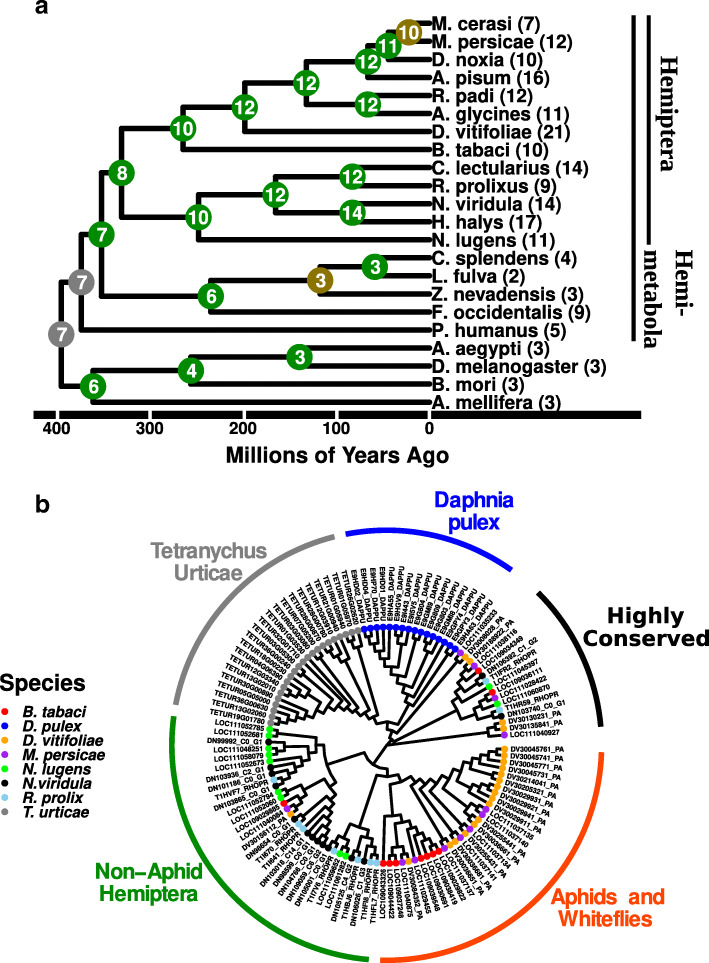
ABC-H family phylogeny in Hemiptera. **a** The evolution within the ABC-H transporter family was analyzed with CAFE. Each tip of the tree represents a species mostly of Hemiptera. The numbers present next to each tip correspond to the number of predicted ABC-H transporters, while the node numbers correspond to CAFE predictions for family sizes. Color coding of the nodes signifies bootstrap support with > 90 % = Green; 70–90 % =Yellow; <70 % = Red; NA = Gray. **b** A phylogeny of a subset of ABC-H transporters was made so as to understand the relationship of these transporters among Hemiptera and other groups. ABC-H proteins appear to group based on lineage, suggesting that multiple expansions occurred during the evolution of Arthropods

Next, we considered the higher number of ABC-H transporters , which have a less defined physiological role. While CAFE found an elevated level of ABC-H transporters in Hemiptera compared to other arthropods, it also suggested that the lineage containing aphids and whiteflies (Sternorrhyncha such as *M. persicae, A. pisum* and *B. tabaci*) and the branch containing other Hemiptera (stink bugs, planthoppers etc. such as *N. viridula, R. prolixus*, and *N. lugens*), may have underwent additional expansions specific to each lineage (Fig. 5A). To address this question, a phylogenetic tree was generated from all ABC-H genes from 6 Hemiptera along with *T. urticae* and *D. pulex* as outgroups. A subset of these ABC-H transporters orthologous to the *Drosophila* gene *Snu* clustered as 1:1 orthologues as reported previously [6]⁠. However, the ABC-H proteins from *D. pulex* and *T. urticae* grouped completely independently, suggesting independent expansions of the ABC-H family in each of these two species. Likewise, most non-*Snu* ABC-H transporters from Sternorrhyncha (aphids and whiteflies such as *M. persicae* and *D. vitifoliae*) clustered together and formed a sister group to the majority of other Hemiptera genes which formed an almost completely monophyletic cluster of ABC-H transporters (Fig. 5B). The independent grouping of Sternorrhyncha and non-Sternorrhyncha ABC-H genes suggests that while the ABC-H family is larger in all Hemiptera, it may have undergone multiple expansions at different times in the evolution of this clade.

**Fig. 6:**
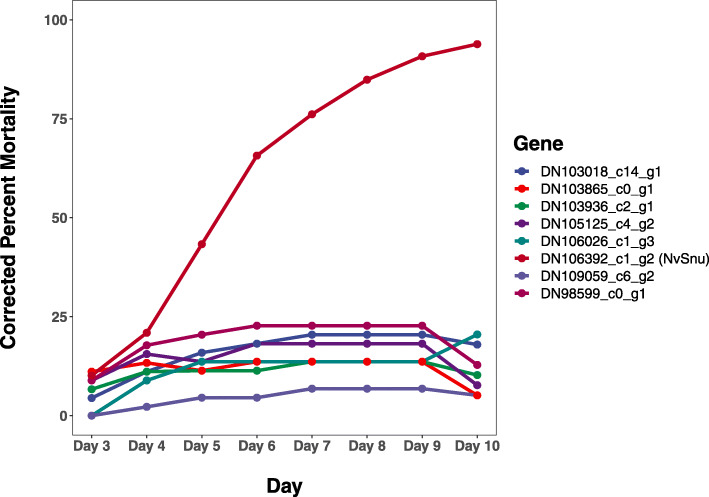
RNAi lethality of ABC-H transporters. The essentiality of the ABC-H transporters was examined in *N. viridula* using RNAi. Following dsRNA injection, mortality was measured from 3 to 10 days. The x axis represents time in days, and the y-axis represents mortality corrected to dsLacZ injected individuals. Only the *Snu* gene gave substantial amounts of mortality, while all other ABC-H genes displayed mortality < 30 %

To study the potential roles of hemipteran specific ABC-H transporters, we used RNAi to individually knock down the expression of several ABC-H genes in the stink bug *N. viridula*. Using previous transcriptomic information [47]⁠, it was observed that the ABC-H genes were expressed primarily in the M4 midgut region and non-gut tissue (Table S7). We selected 8 out of 14 genes for RNAi knockdown based on high expression levels. Significant reduction in expression levels ranging from 70 to 97 % was observed for 5 out of the 8 genes following knockdown attempts (Figure S5). No observable phenotype was detected in knockdown individuals apart from the constructs targeting the gene homologous to *D. melanogaster Snu* (*DN106392_c1_g2)*, which led to mortality in almost all nymphs 10 days post injection (Fig. 6). A shriveling of the body was observed in these nymphs suggesting that desiccation may underpin this mortality. The *Snu* gene is highly conserved appearing as a 1:1 orthologue in all Hemiptera species examined and it is closely related to the *Snu* gene previously studied in other insects (see discussion).

## Discussion

There has been substantial interest in the ABC superfamily among arthropods both as mediators of pesticide resistance and as targets for pest control. Their status as one of the largest and best understood gene superfamilies also make them a highly interesting evolutionary model. The increased rate of genome and transcriptome sequencing in recent years has made it possible to observe how such superfamilies evolve on various evolutionary timescales using comparative genomics approaches. While several studies have performed annotations in individual species or looked at closely related species (See references in Table S4), there has been few comparative genomic studies of ABC evolution across arthropods. The two exceptions were Dermauw et al. [6], which used a small number of species and Rane et al. [28] which, only looked at total ABC number rather than specific family evolution.

### The ABC_scan pipeline

In this study, we implemented an algorithm to identify ABC transporters from the predicted protein set of non-model arthropods. The algorithm performs well (< 10 % error) when benchmarked against manually curated datasets in other species (Figure S2), and it appears that most differences centered around different starting protein sets and our decision to remove short amino acid fragments under 250 residues. Because *ABC_scan* represented a reasonably robust tool to catalogue and sort ABC transporters, a standalone executable version of ABC_scan (https://github.com/shanedenecke/ABC_scan.git) and web application (http://chrysalida.imbb.forth.gr:3838/ABC_scan/) were designed to annotate new protein sets. This appears to be of interest as there are a substantial number of studies that are composed almost exclusively of such annotations with often less comprehensive identification pipelines. As the pipeline is generic apart from the use of several arthropod specific ABC sets in order to sort candidate transporters, it could theoretically be adapted to non-arthropod species, although this was not tested in this study.

However, there are several limitations to *ABC_scan*. Because it searches amino acid sequences, poor genome annotations will under or overestimate ABC gene counts (hence the BUSCO threshold used here). Furthermore, manual curation will almost always be able to provide more reliable estimates and fine tune gene models. Still, the accessibility of *ABC_scan* and the fact that it is more comprehensive than many published methodologies suggests that it might be of use to the wider research community occupying a space between detailed manual curation and completely automated annotations from larger databases (e.g. NCBI).

### The expansion of xenobiotic transporting genes in Coleoptera and Lepidoptera

Deploying *ABC_scan* to search 158 non-model arthropods identified several interesting evolutionary trends in the ABC-C and ABC-BF families previously associated with xenobiotic transport. The ABC-C family was not the subject of intense focus in this study but showed a clear expansion in polyphagous beetles (Cucujiformia) compared to other beetles and non-beetle insects (Figure S4). A recent study implicated members of this ABC-C expansion in pesticide resistance, which highlights the adaptive potential of recent gene family expansions [48]⁠. A significant expansion of the ABC-BF in Lepidoptera was also detected and seems to have occurred soon after the divergence of this order (Fig. 5). This expansion runs in parallel to a similar expansion of the SLC22 (organic ion) transporter family in this group, which has also been associated with xenobiotic transport [27]⁠. The expansion of these “detoxification” families within insect lineages may also suggest adaptations to a more complex diet of plant materials, but functional work will be needed to confirm or reject this hypothesis and implicate individual genes.

Characterization of the expression patterns of the ABCB full transporter family in *H. armigera* yielded interesting results. Despite residing in a genomic cluster, *HaABC-B1, HaABC-B2* and *HaABC-B3* showed relatively divergent expression patterns (Fig. 4). A Similar situation was observed in the leaf beetle *Chrysochus auratus* where three P-glycoprotein paralogues at the same genomic locus display strong differences in their expression patterns [49]⁠. The paralogues in *D. melanogaster* also show such partitioning among its four P-gp paralogues [15]. In the *H. armigera* central nervous system the *HaABC-B1* gene predominated, and it is likely this localizes to the blood brain barrier, acting as a xenobiotic transporter similar to the case in other insects and humans [12, 50]⁠. Similarly, the midgut expressed only one transporter, *HaABC-B7*, which also showed abundant expression in the Malpighian tubules (Table S6). In other insects such as *Plutella xylostella*, *L. decemlineata* and *Trichoplusia ni* the expression of P-gp orthologues was enriched in the midgut and Malpighian tubules [51–53]⁠, and similar results were also obtained from a study of *H. armigera* transporters with qPCR [54]⁠. Further work will be needed to fully understand the relationship between expression patterns of P-glycoprotein paralogues within and between species. Additional functional work is also necessary. A recent study showed that CRISPR-Cas9 mediated knockout of another Lepidoptera *Spodoptera exigua* increased sensitivity to emamectin benzoate [55]. Of all the P-glycoproteins in *H. armigera, HaABC-B7* has underwent functional characterization as a purified protein [56]⁠, and a recent CRISPR mediated knockout of *HaABC-B6* increased sensitivity to the plant secondary metabolite gossypol [57]⁠.

### RNAi knockdown of ABC-H transporters in N. viridula

The discovery of an enrichment of ABC-H transporters in Hemiptera prompted us to examine what physiological roles these transporters play. To the authors knowledge, this study is the first attempt to study ABC-H genes in Hemiptera. From the phylogenetic analysis it appears that there were independent expansions of ABC-H transporters among Sternorrhyncha (aphids and whiteflies) and non-Sternorrhyncha, along with a more conserved cluster of 1:1 orthologues (Fig. 5B). Many of these transporters were expressed in the M4 region which is divergent from the rest of the midgut and is thought to play a role in harboring symbiotic microorganisms [58]. However, the functional role these transporters play in this tissue is not clear.

Knockdown of the more conserved *DN106392_C1_G2*, induced a high level of mortality and nymphs appeared to desiccate or die during the transition from the 2nd to the 3rd nymphal stage (Fig. 6). This gene is also the most closely related to *Snu*, which has been characterized as an essential gene in cuticular formation through hydrocarbon transport at the epidermis [20–22]⁠. In contrast, no observable phenotype was detected when the hemipteran specific ABC-H genes were targeted with dsRNA despite a successful knockdown. While this may be due to the technical limitations of RNAi, one explanation may be that the enrichment of ABC-H transporters in Hemiptera has resulted in functional redundancy between the transporters of this family. The evolutionary reason for this gene expansion in Hemiptera will require in-depth molecular studies of the physiological role of these so far largely unexplored ABC-H transporter family.

## Supplementary Information

**Additional file 1: Figure S1. **The total number of ABC transporters in each species (x-axis) was calculated for each species included in the analysis.

**Additional file 2: Figure S2. **The predicted numbers of ABC transporters in the ABC_scan pipeline were benchmarked against previously published ABC transporter datasets. The % difference in total number of ABC transporters (y-axis) was plotted for each species (x-axis). For all species tested, the difference was less than 10% (dotted line).

**Additional file 3: Figure S3. **A comparison was made among the non-variable ABC families (ABC-BH, ABC-D, ABC-E, and ABC-F) with family size (y-axis) broken down by both taxonomic order (x-axis) and family (panels). Orders are color coded, and boxplots include a horizontal black bar for median, boxes for upper quartiles, dots for outliers. Lower case letters above the boxes signify statistical groups generated by the Kruskal-Wallis test.

**Additional file 4: Figure S4. **A CAFE analysis for ABC-C proteins was performed with a focus on Coleoptera. Higher numbers of ABC-C proteins were observed in Cucujiformia beetles (e.g. *T. castaneum, L. decemlineata*) compared to other beetles and non-beetle arthropods. The numbers present next to each tip correspond to the number of predicted ABC-C transporters, while the node numbers correspond to CAFE predictions for ABC-B full transporter family sizes. Color coding of the nodes signifies bootstrap support with >90 % percent = Green; 70-90% =Yellow; <70% = Red; NA=Gray.

**Additional file 5: Figure S5. **qPCR was performed for all ABC-H genes knocked down in *N. viridula*. For each gene at least 3 biological replicates were performed, and all values were calculated using the ΔΔCt method and normalized to 1.

**Additional file 6: Table S1. **This table shows all the metadata associated with every species included in the analysis. ABC counts are missing for species with a BUSCO scores of below %80 Single copy orthologues.

**Additional file 7: Table S2. **Species level phylogenies used in CAFE were constructed with the species shown in this table. Outgroups are highlighted in yellow.

**Additional file 8: Table S3. **All primers used in the *N. viridula* RNAi screen are shown.

**Additional file 9: Table S4. **The raw data for Figure S2 is shown, which contains the total number of ABC transporters identified in *ABC_scan* and the literature for a subset of species. Also included is the source of the literature derived counts.

**Additional file 10: Table S5. **All significant outputs of the Kruskal-Wallis analysis measuring the effect of taxonomic group on ABC family size are listed.

**Additional file 11: Table S6. **A transcriptomic analysis of *H. armigera* midgut, Malpighian tubules and central nervous system was undertaken to examine P-glycoprotein expression in these epithelial tissues. H. armigera genes are listed with their official gene set (OGS) codes, corresponding NCBI accession numbers, and their expression values in each tissue displayed in transcripts per million (TPM).

**Additional file 12: Table S7. **All predicted ABC-H genes in N. viridula are listed along with transcriptomic data derived from [47].

## Data Availability

All data analyzed in this study is publicly available. All analysis scripts can be found on GitHub https://github.com/shanedenecke/ABC_scan.git. The data can also be accessed via a web application at http://chrysalida.imbb.forth.gr:3838/ABC_scan/. Raw sequencing can be found on the sequence read archive at PRJNA719695.
