# Supplementary figures and images for: Comparative and functional genomics of the ABC transporter superfamily across arthropods
Source: BMC Genomics. 2021 Jul 19;22:553. doi: 10.1186/s12864-021-07861-2 (PMC8290562; doi:10.1186/s12864-021-07861-2)

**Frequency**

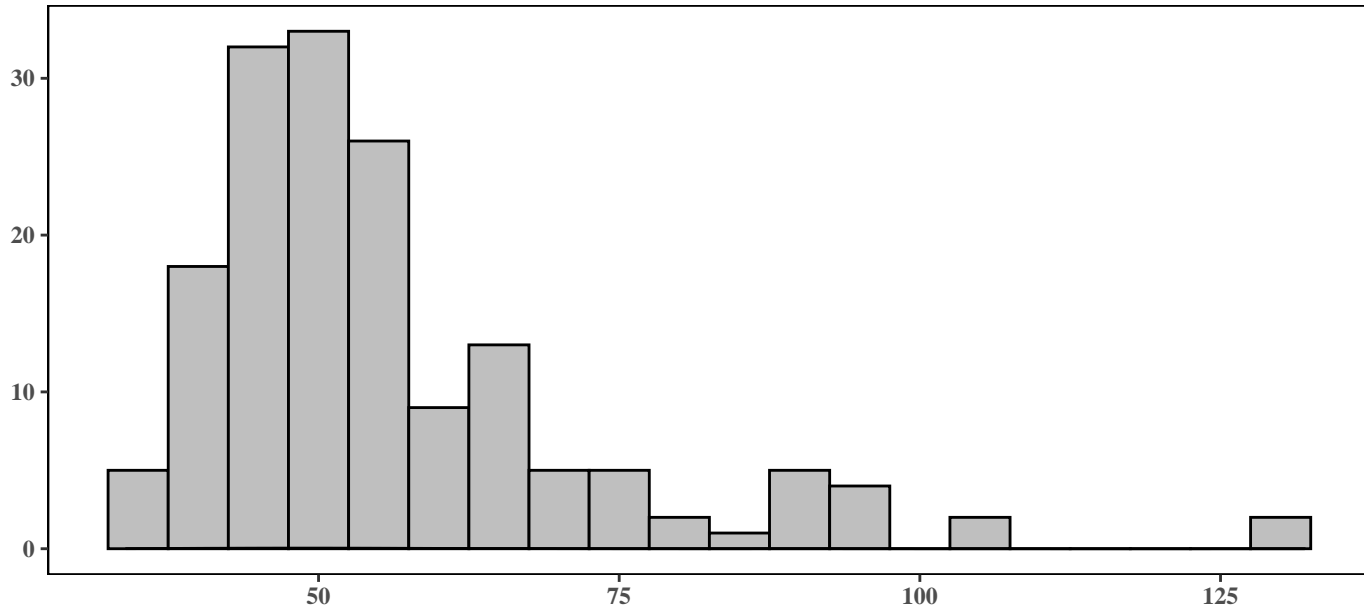

**Total ABCs Identified in Species**

Supplement: Supplementary file 1 — Additional file 1: Figure S1. The total number of ABC transporters in each species (x-axis) was calculated for each species included in the analysis. [file 12864_2021_7861_MOESM1_ESM.pdf]

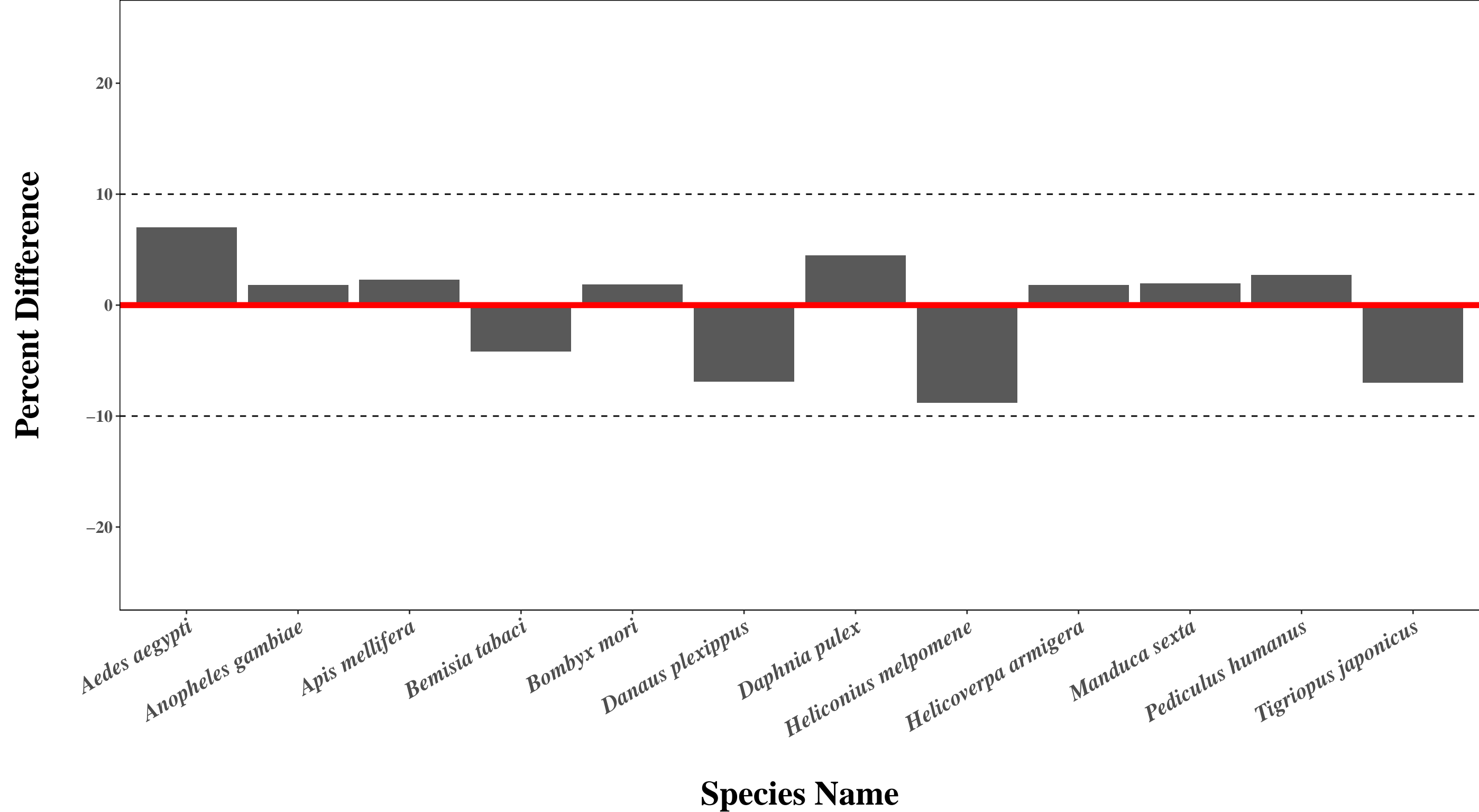

Supplement: Supplementary file 2 — Additional file 2: Figure S2. The predicted numbers of ABC transporters in the ABC_scan pipeline were benchmarked against previously published ABC transporter datasets. The % difference in total number of ABC transporters (y-axis) was plotted for each species (x-axis). For all species tested, the difference was less than 10% (dotted line). [file 12864_2021_7861_MOESM2_ESM.pdf]

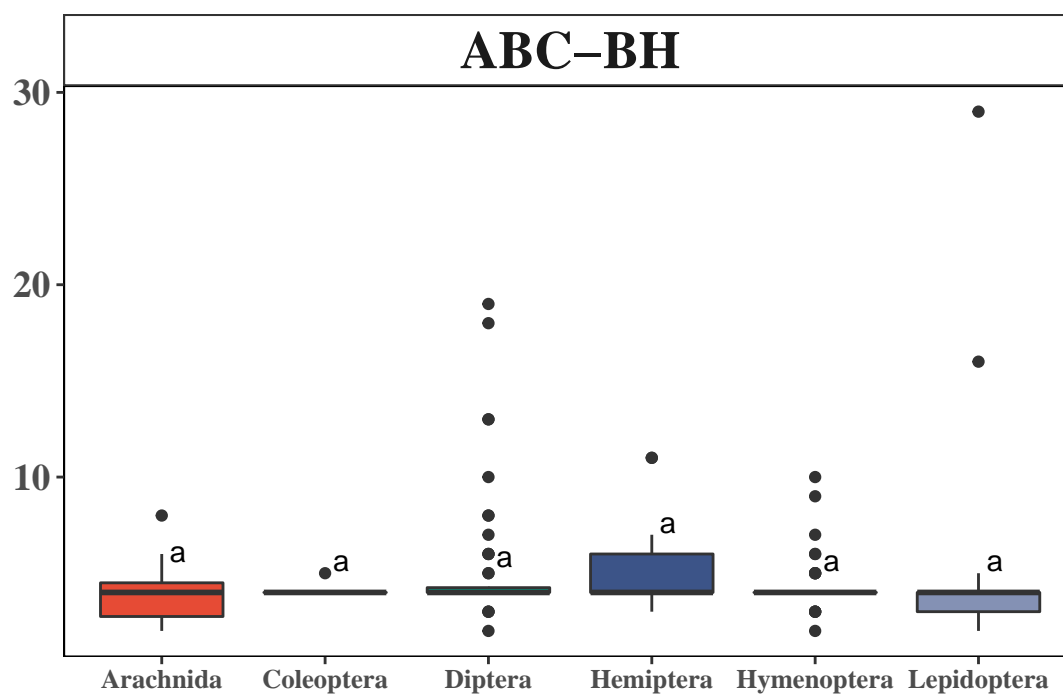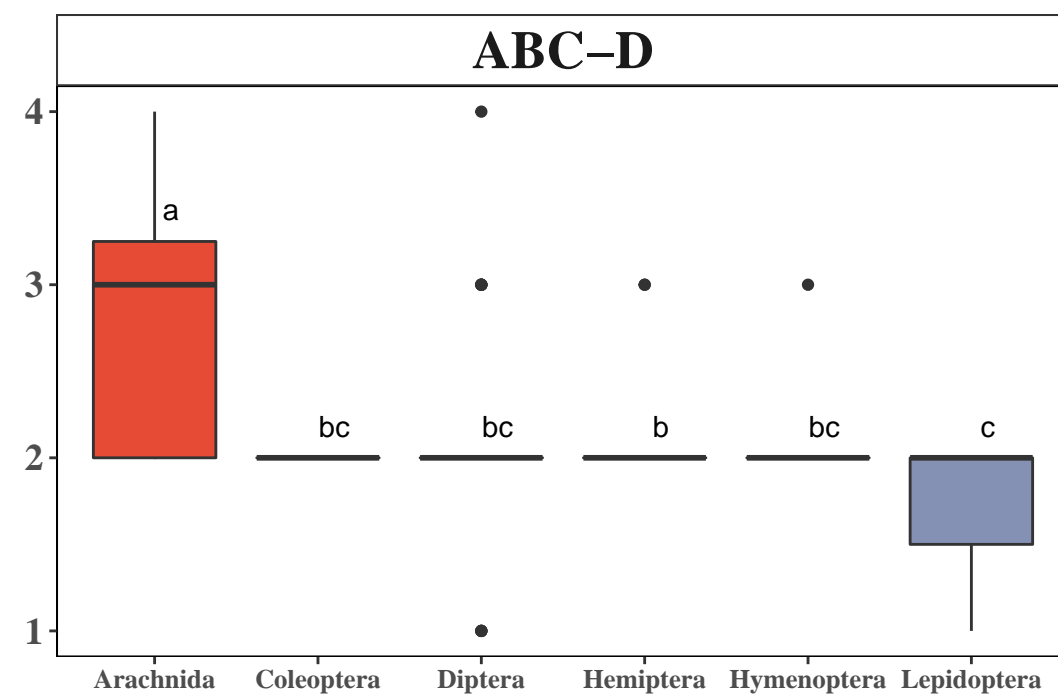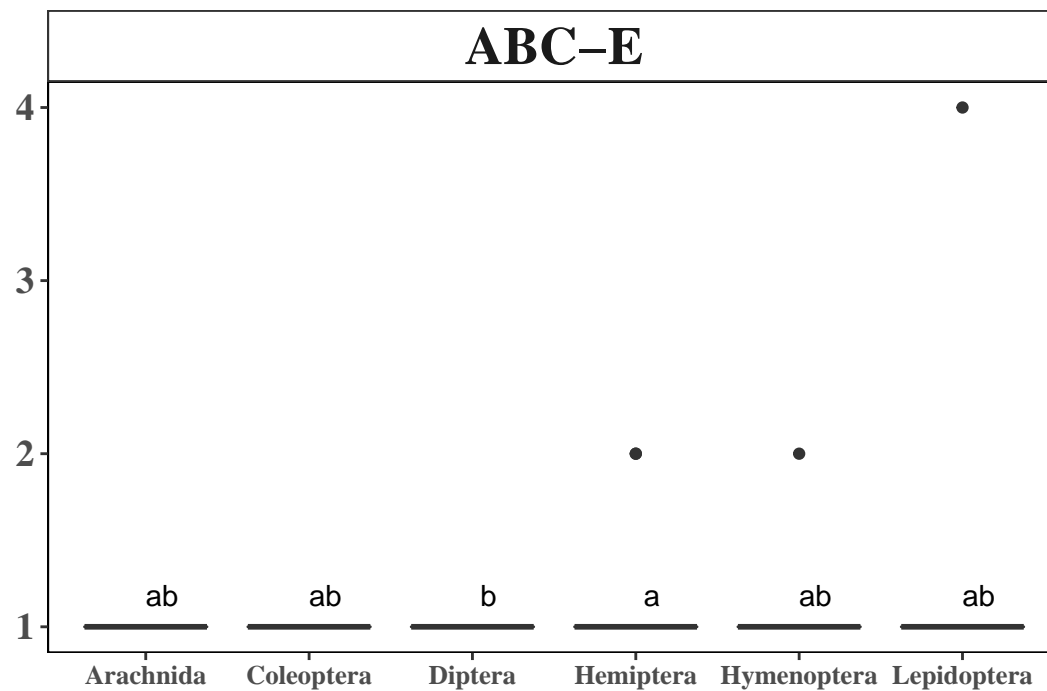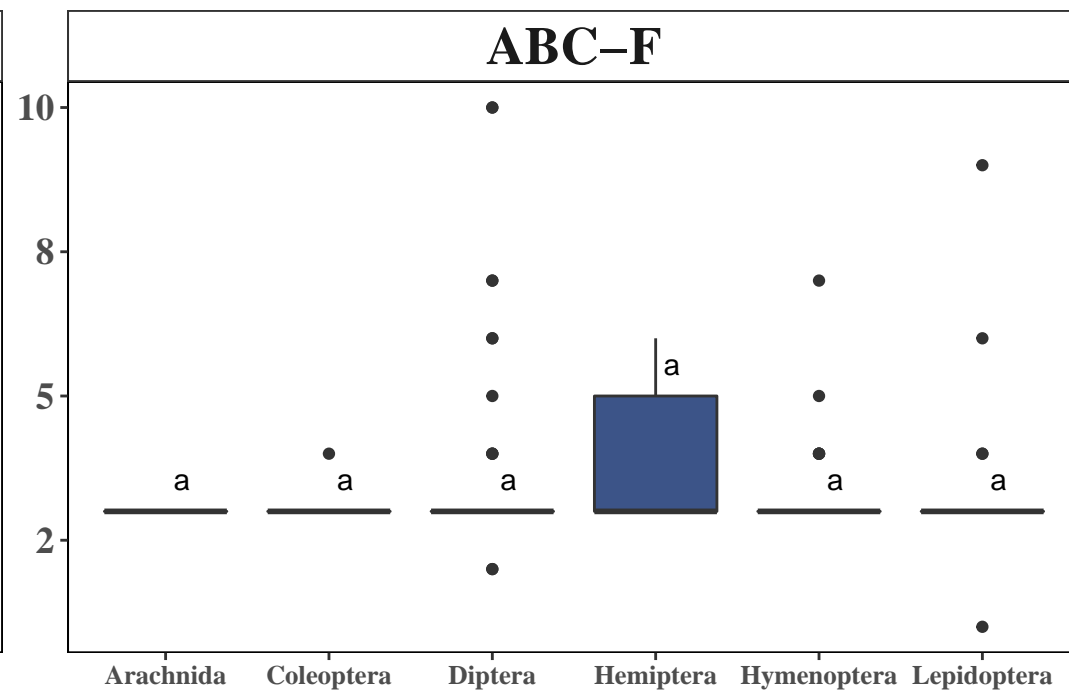

Supplement: Supplementary file 3 — Additional file 3: Figure S3. A comparison was made among the non-variable ABC families (ABC-BH, ABC-D, ABC-E, and ABC-F) with family size (y-axis) broken down by both taxonomic order (x-axis) and family (panels). Orders are color coded, and boxplots include a horizontal black bar for median, boxes for upper quartiles, dots for outliers. Lower case letters above the boxes signify statistical groups generated by the Kruskal-Wallis test. [file 12864_2021_7861_MOESM3_ESM.pdf]

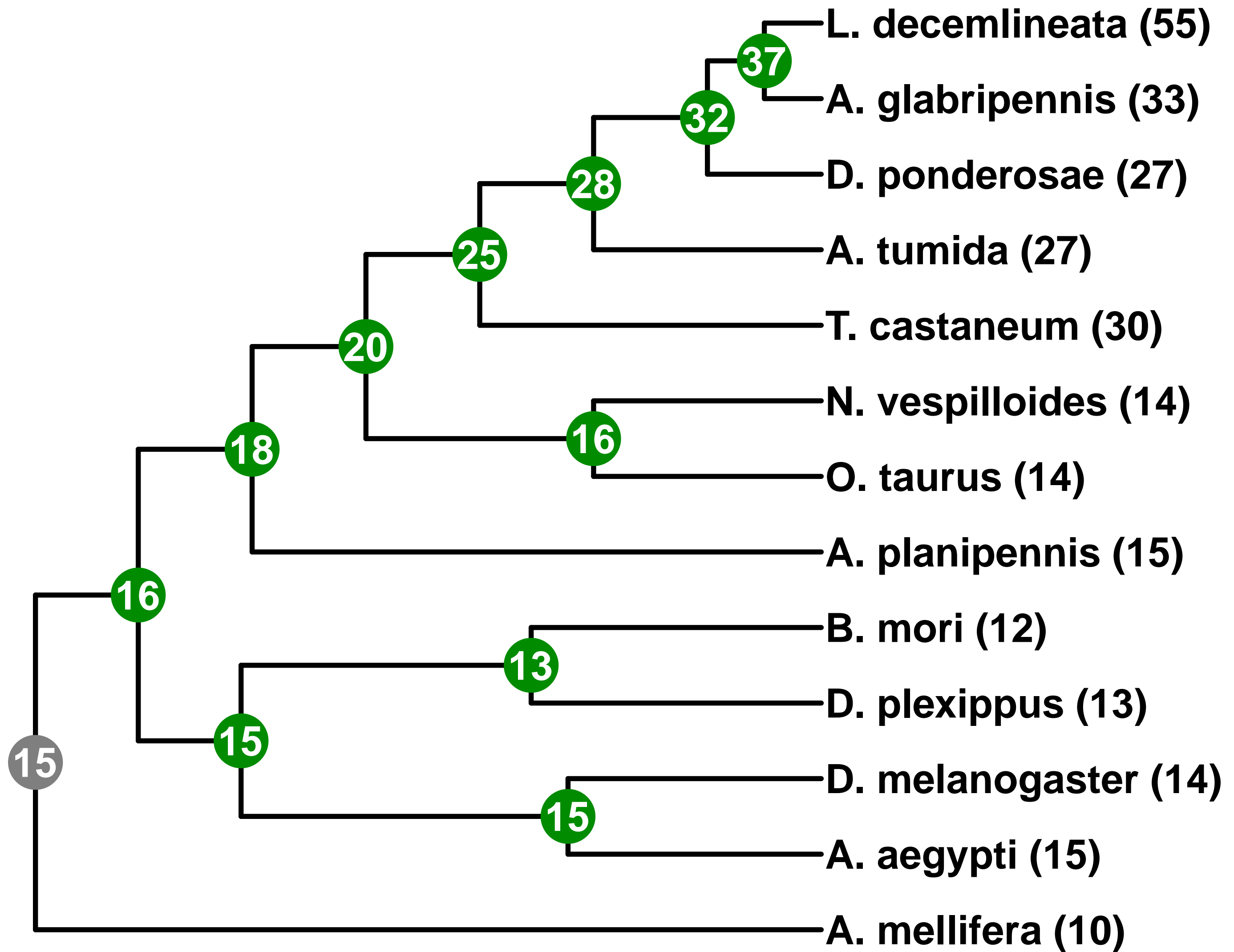

Supplement: Supplementary file 4 — Additional file 4: Figure S4. A CAFE analysis for ABC-C proteins was performed with a focus on Coleoptera. Higher numbers of ABC-C proteins were observed in Cucujiformia beetles (e.g. T. castaneum, L. decemlineata) compared to other beetles and non-beetle arthropods. The numbers present next to each tip correspond to the number of predicted ABC-C transporters, while the node numbers correspond to CAFE predictions for ABC-B full transporter family sizes. Color coding of the nodes signifies bootstrap support with >90 % percent = Green; 70-90% =Yellow; <70% = Red; NA=Gray. [file 12864_2021_7861_MOESM4_ESM.pdf]

power\_combined2

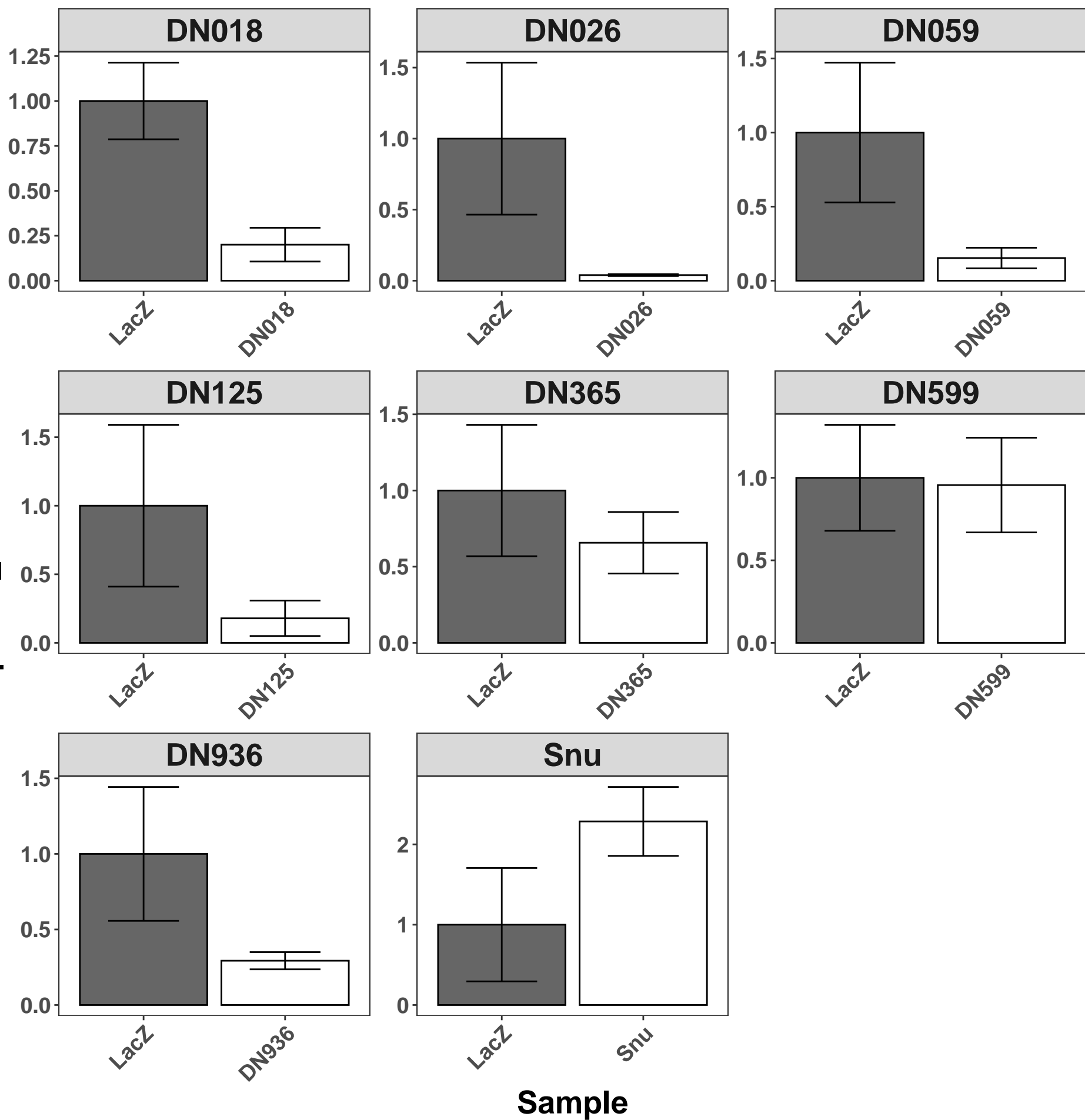

Supplement: Supplementary file 5 — Additional file 5: Figure S5. qPCR was performed for all ABC-H genes knocked down in N. viridula. For each gene at least 3 biological replicates were performed, and all values were calculated using the ΔΔCt method and normalized to 1. [file 12864_2021_7861_MOESM5_ESM.pdf]
